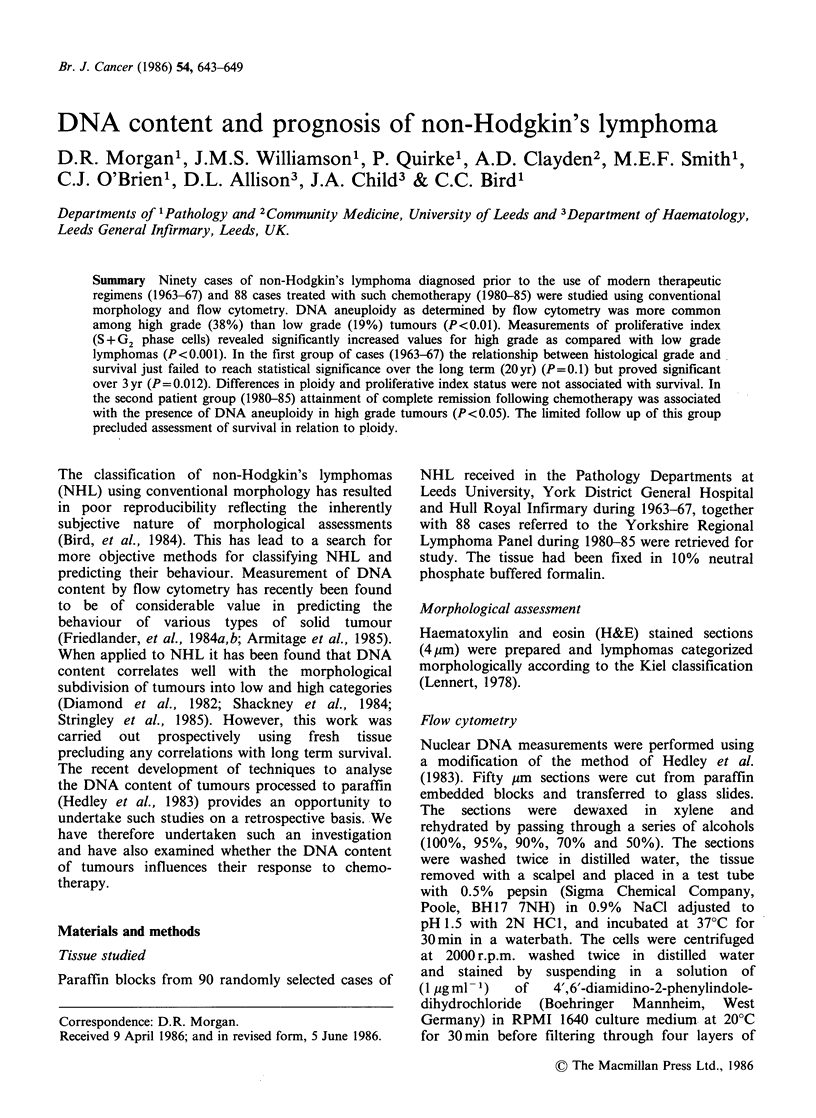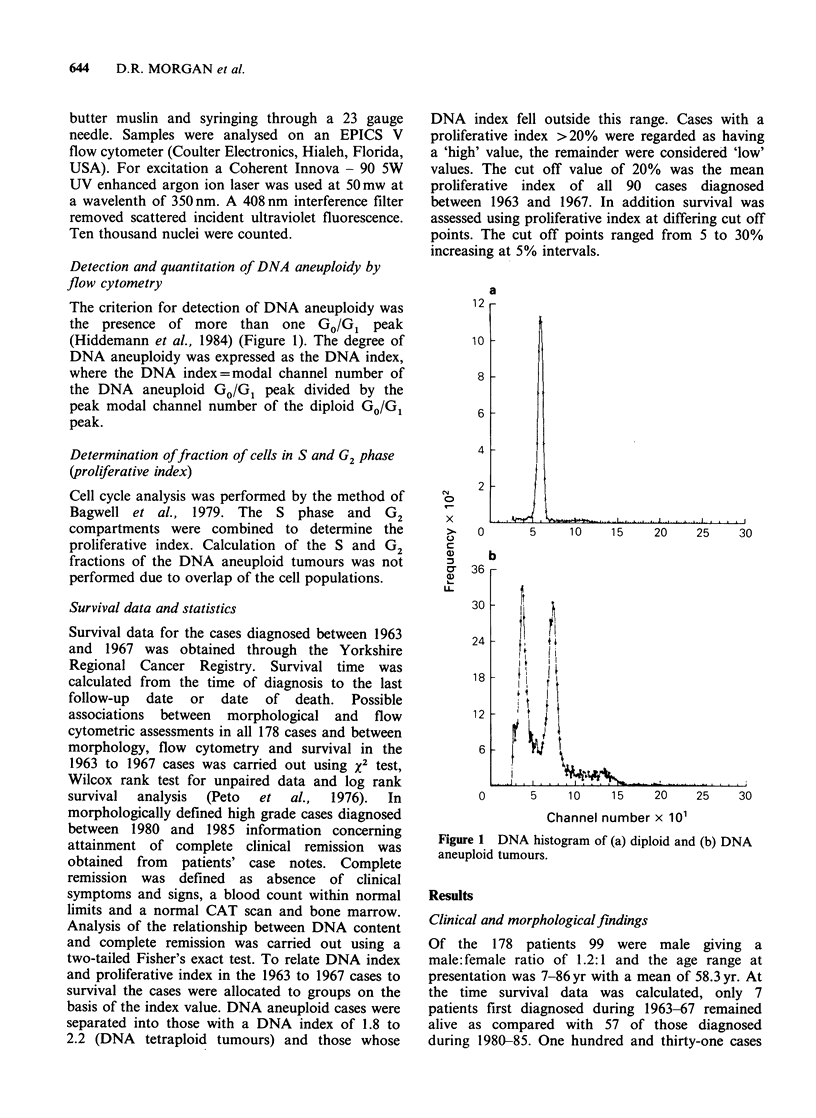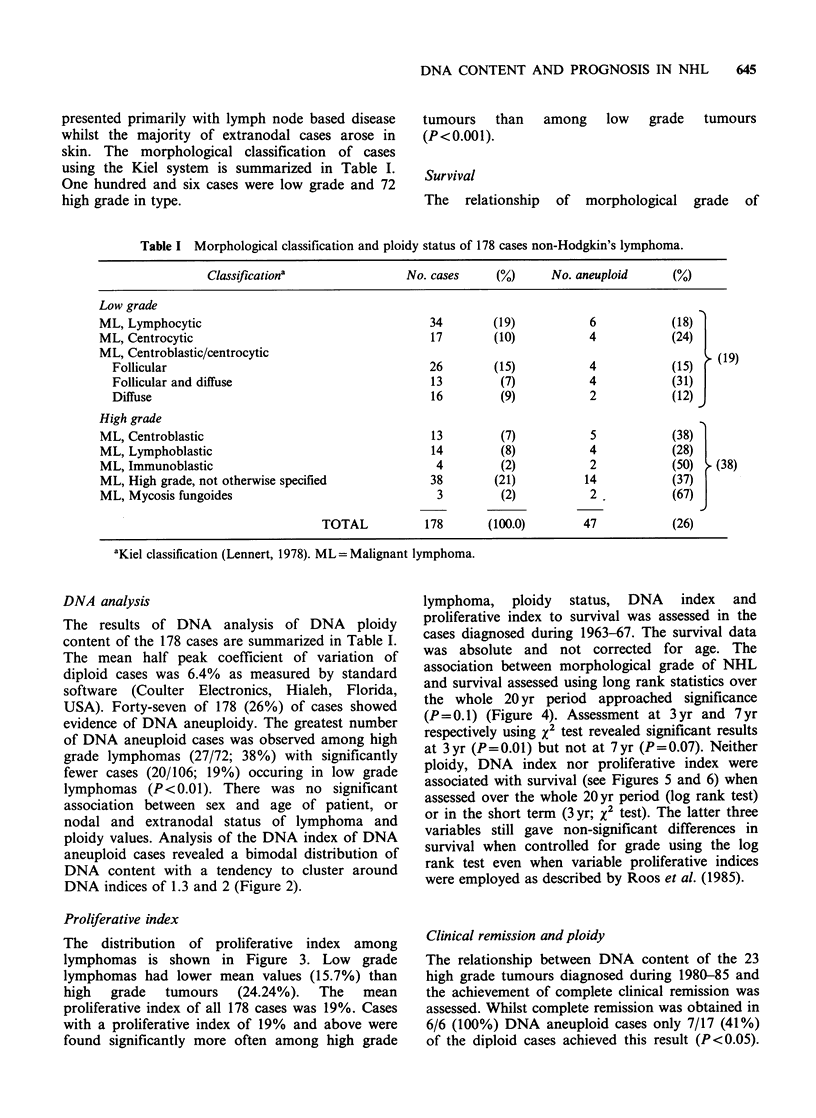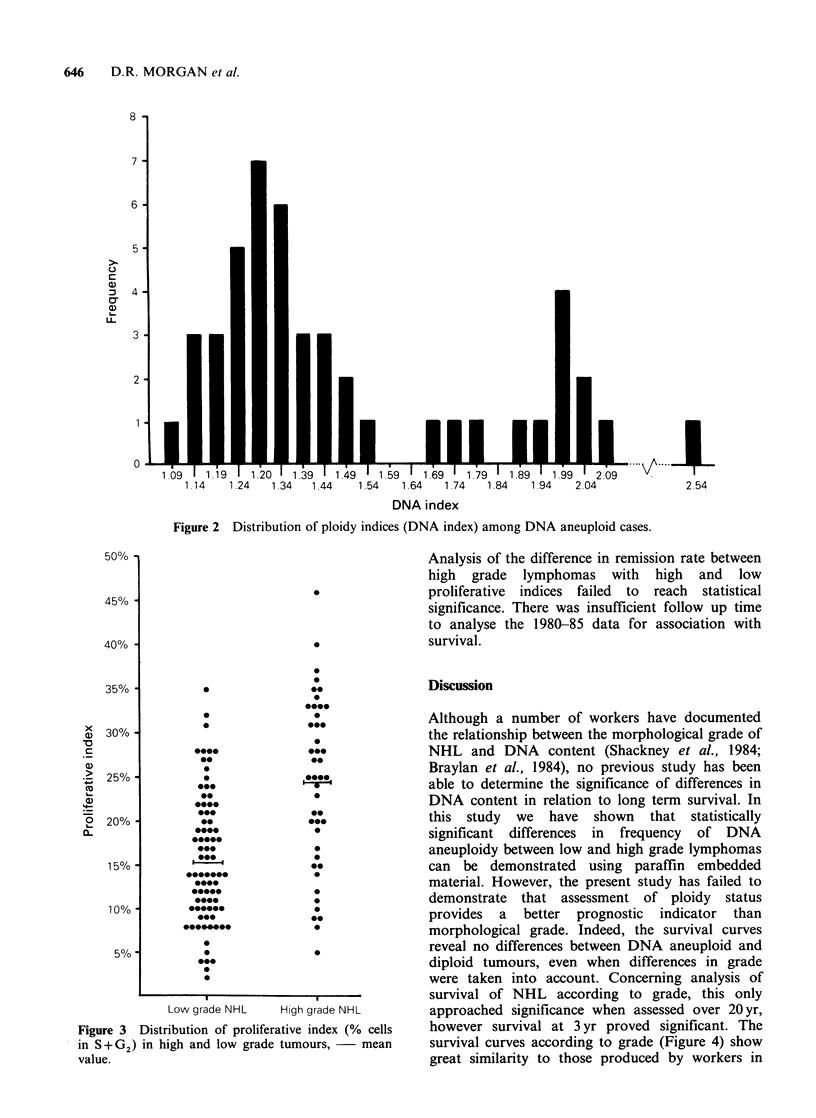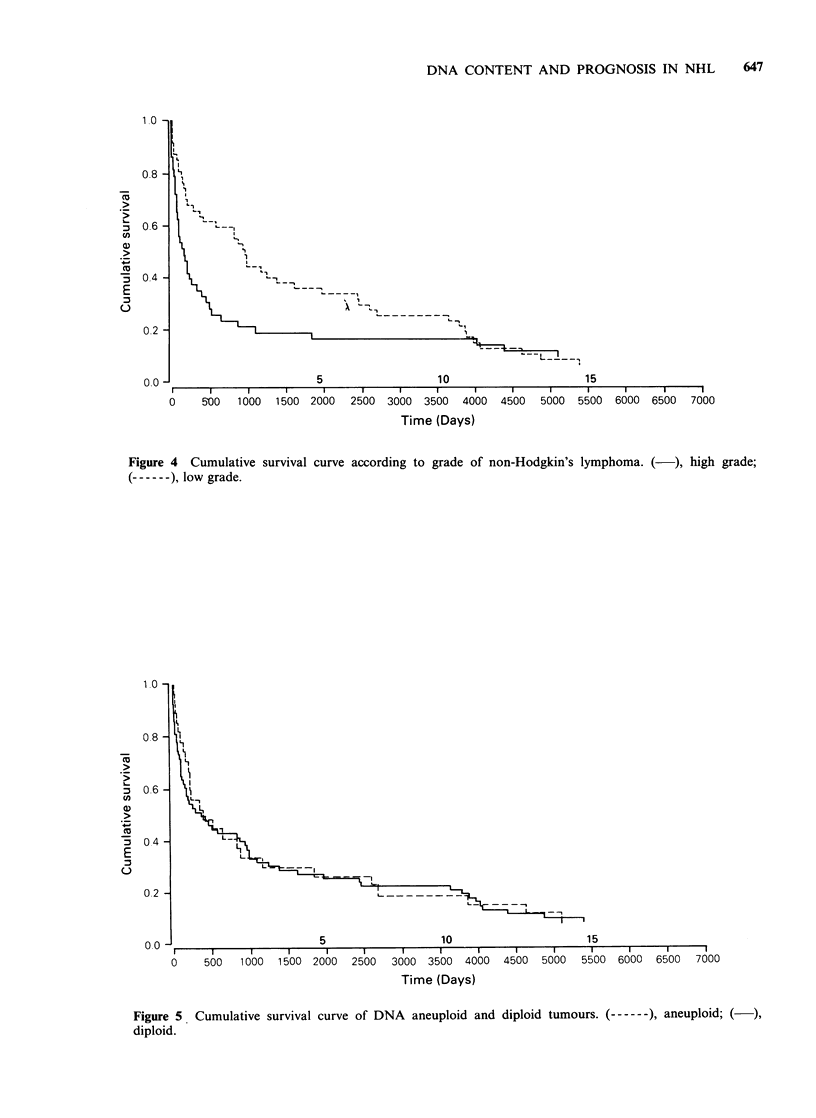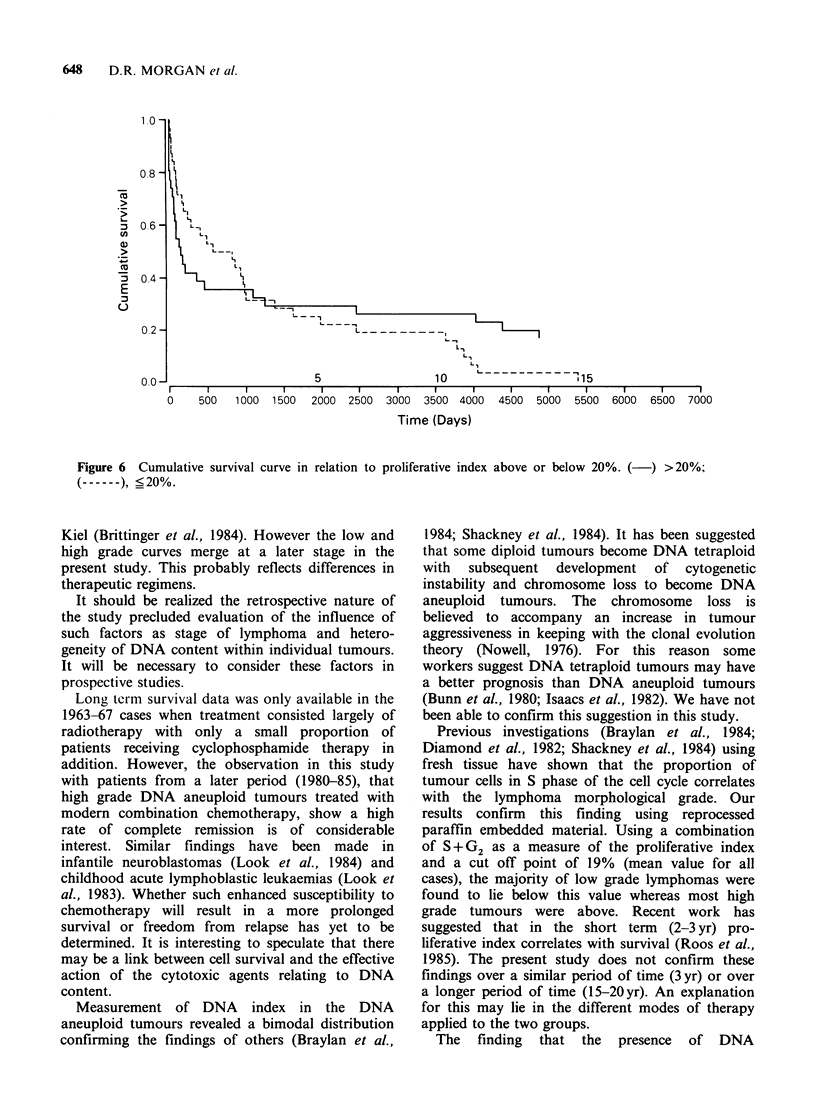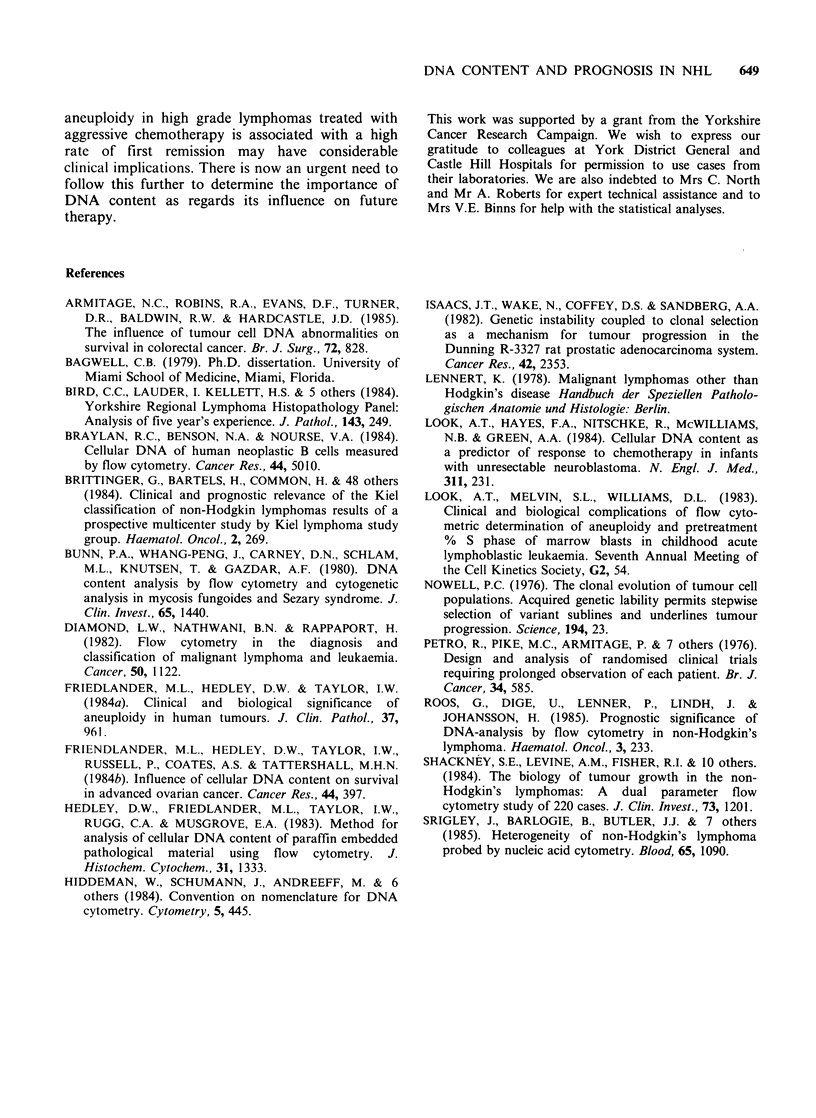# DNA content and prognosis of non-Hodgkin's lymphoma.

**DOI:** 10.1038/bjc.1986.221

**Published:** 1986-10

**Authors:** D. R. Morgan, J. M. Williamson, P. Quirke, A. D. Clayden, M. E. Smith, C. J. O'Brien, D. L. Allison, J. A. Child, C. C. Bird

## Abstract

Ninety cases of non-Hodgkin's lymphoma diagnosed prior to the use of modern therapeutic regimens (1963-67) and 88 cases treated with such chemotherapy (1980-85) were studied using conventional morphology and flow cytometry. DNA aneuploidy as determined by flow cytometry was more common among high grade (38%) than low grade (19%) tumours (P less than 0.01). Measurements of proliferative index (S + G2 phase cells) revealed significantly increased values for high grade as compared with low grade lymphomas (P less than 0.001). In the first group of cases (1963-67) the relationship between histological grade and survival just failed to reach statistical significance over the long term (20 yr) (P = 0.1) but proved significant over 3 yr (P = 0.012). Differences in ploidy and proliferative index status were not associated with survival. In the second patient group (1980-85) attainment of complete remission following chemotherapy was associated with the presence of DNA aneuploidy in high grade tumours (P less than 0.05). The limited follow up of this group precluded assessment of survival in relation to ploidy.


*Br. J. Cancer* (1986) **54**, 643–649

### DNA content and prognosis of non-Hodgkin's lymphoma

D.R. Morgan[1], J.M.S. Williamson[1], P. Quirke[1], A.D. Clayden[2], M.E.F. Smith[1], C.J. O'Brien[1], D.L. Allison[3], J.A. Child[3] & C.C. Bird[1]

*Departments of [1]Pathology and [2]Community Medicine, University of Leeds and [3]Department of Haematology, Leeds General Infirmary, Leeds, UK.*

**Summary** Ninety cases of non-Hodgkin's lymphoma diagnosed prior to the use of modern therapeutic regimens (1963–67) and 88 cases treated with such chemotherapy (1980–85) were studied using conventional morphology and flow cytometry. DNA aneuploidy as determined by flow cytometry was more common among high grade (38%) than low grade (19%) tumours ($P<0.01$). Measurements of proliferative index ($S+G_2$ phase cells) revealed significantly increased values for high grade as compared with low grade lymphomas ($P<0.001$). In the first group of cases (1963–67) the relationship between histological grade and survival just failed to reach statistical significance over the long term (20 yr) ($P=0.1$) but proved significant over 3 yr ($P=0.012$). Differences in ploidy and proliferative index status were not associated with survival. In the second patient group (1980–85) attainment of complete remission following chemotherapy was associated with the presence of DNA aneuploidy in high grade tumours ($P<0.05$). The limited follow up of this group precluded assessment of survival in relation to ploidy.

The classification of non-Hodgkin's lymphomas (NHL) using conventional morphology has resulted in poor reproducibility reflecting the inherently subjective nature of morphological assessments (Bird, *et al.*, 1984). This has lead to a search for more objective methods for classifying NHL and predicting their behaviour. Measurement of DNA content by flow cytometry has recently been found to be of considerable value in predicting the behaviour of various types of solid tumour (Friedlander, *et al.*, 1984*a,b*; Armitage *et al.*, 1985). When applied to NHL it has been found that DNA content correlates well with the morphological subdivision of tumours into low and high categories (Diamond *et al.*, 1982; Shackney *et al.*, 1984; Stringley *et al.*, 1985). However, this work was carried out prospectively using fresh tissue precluding any correlations with long term survival. The recent development of techniques to analyse the DNA content of tumours processed to paraffin (Hedley *et al.*, 1983) provides an opportunity to undertake such studies on a retrospective basis. We have therefore undertaken such an investigation and have also examined whether the DNA content of tumours influences their response to chemotherapy.

### Materials and methods

### *Tissue studied*

Paraffin blocks from 90 randomly selected cases of NHL received in the Pathology Departments at Leeds University, York District General Hospital and Hull Royal Infirmary during 1963–67, together with 88 cases referred to the Yorkshire Regional Lymphoma Panel during 1980–85 were retrieved for study. The tissue had been fixed in 10% neutral phosphate buffered formalin.

### *Morphological assessment*

Haematoxylin and eosin (H&E) stained sections (4 $\mu$m) were prepared and lymphomas categorized morphologically according to the Kiel classification (Lennert, 1978).

### *Flow cytometry*

Nuclear DNA measurements were performed using a modification of the method of Hedley *et al.* (1983). Fifty $\mu$m sections were cut from paraffin embedded blocks and transferred to glass slides. The sections were dewaxed in xylene and rehydrated by passing through a series of alcohols (100%, 95%, 90%, 70% and 50%). The sections were washed twice in distilled water, the tissue removed with a scalpel and placed in a test tube with 0.5% pepsin (Sigma Chemical Company, Poole, BH17 7NH) in 0.9% NaCl adjusted to pH 1.5 with 2N HCl, and incubated at 37°C for 30 min in a waterbath. The cells were centrifuged at 2000 r.p.m. washed twice in distilled water and stained by suspending in a solution of ($1\,\mu g\,ml^{-1}$) of 4′,6′-diamidino-2-phenylindole-dihydrochloride (Boehringer Mannheim, West Germany) in RPMI 1640 culture medium at 20°C for 30 min before filtering through four layers of

Correspondence: D.R. Morgan.
Received 9 April 1986; and in revised form, 5 June 1986.

© The Macmillan Press Ltd., 1986

butter muslin and syringing through a 23 gauge needle. Samples were analysed on an EPICS V flow cytometer (Coulter Electronics, Hialeh, Florida, USA). For excitation a Coherent Innova - 90 5W UV enhanced argon ion laser was used at 50 mw at a wavelenth of 350 nm. A 408 nm interference filter removed scattered incident ultraviolet fluorescence. Ten thousand nuclei were counted.

*Detection and quantitation of DNA aneuploidy by flow cytometry*

The criterion for detection of DNA aneuploidy was the presence of more than one $G_0/G_1$ peak (Hiddemann *et al.*, 1984) (Figure 1). The degree of DNA aneuploidy was expressed as the DNA index, where the DNA index = modal channel number of the DNA aneuploid $G_0/G_1$ peak divided by the peak modal channel number of the diploid $G_0/G_1$ peak.

*Determination of fraction of cells in S and $G_2$ phase (proliferative index)*

Cell cycle analysis was performed by the method of Bagwell *et al.*, 1979. The S phase and $G_2$ compartments were combined to determine the proliferative index. Calculation of the S and $G_2$ fractions of the DNA aneuploid tumours was not performed due to overlap of the cell populations.

*Survival data and statistics*

Survival data for the cases diagnosed between 1963 and 1967 was obtained through the Yorkshire Regional Cancer Registry. Survival time was calculated from the time of diagnosis to the last follow-up date or date of death. Possible associations between morphological and flow cytometric assessments in all 178 cases and between morphology, flow cytometry and survival in the 1963 to 1967 cases was carried out using $\chi^2$ test, Wilcox rank test for unpaired data and log rank survival analysis (Peto *et al.*, 1976). In morphologically defined high grade cases diagnosed between 1980 and 1985 information concerning attainment of complete clinical remission was obtained from patients' case notes. Complete remission was defined as absence of clinical symptoms and signs, a blood count within normal limits and a normal CAT scan and bone marrow. Analysis of the relationship between DNA content and complete remission was carried out using a two-tailed Fisher's exact test. To relate DNA index and proliferative index in the 1963 to 1967 cases to survival the cases were allocated to groups on the basis of the index value. DNA aneuploid cases were separated into those with a DNA index of 1.8 to 2.2 (DNA tetraploid tumours) and those whose DNA index fell outside this range. Cases with a proliferative index >20% were regarded as having a 'high' value, the remainder were considered 'low' values. The cut off value of 20% was the mean proliferative index of all 90 cases diagnosed between 1963 and 1967. In addition survival was assessed using proliferative index at differing cut off points. The cut off points ranged from 5 to 30% increasing at 5% intervals.

**Figure 1** DNA histogram of (a) diploid and (b) DNA aneuploid tumours.

### Results

*Clinical and morphological findings*

Of the 178 patients 99 were male giving a male:female ratio of 1.2:1 and the age range at presentation was 7–86 yr with a mean of 58.3 yr. At the time survival data was calculated, only 7 patients first diagnosed during 1963–67 remained alive as compared with 57 of those diagnosed during 1980–85. One hundred and thirty-one cases presented primarily with lymph node based disease whilst the majority of extranodal cases arose in skin. The morphological classification of cases using the Kiel system is summarized in Table I. One hundred and six cases were low grade and 72 high grade in type.

**Table I** Morphological classification and ploidy status of 178 cases non-Hodgkin's lymphoma.

| *Classification*[a] | *No. cases* | (%) | *No. aneuploid* | (%) | |
|---|---|---|---|---|---|
| *Low grade* | | | | | |
| ML, Lymphocytic | 34 | (19) | 6 | (18) | |
| ML, Centrocytic | 17 | (10) | 4 | (24) | |
| ML, Centroblastic/centrocytic | | | | | |
| Follicular | 26 | (15) | 4 | (15) | (19) |
| Follicular and diffuse | 13 | (7) | 4 | (31) | |
| Diffuse | 16 | (9) | 2 | (12) | |
| *High grade* | | | | | |
| ML, Centroblastic | 13 | (7) | 5 | (38) | |
| ML, Lymphoblastic | 14 | (8) | 4 | (28) | |
| ML, Immunoblastic | 4 | (2) | 2 | (50) | (38) |
| ML, High grade, not otherwise specified | 38 | (21) | 14 | (37) | |
| ML, Mycosis fungoides | 3 | (2) | 2 | (67) | |
| TOTAL | 178 | (100.0) | 47 | (26) | |

[a]Kiel classification (Lennert, 1978). ML = Malignant lymphoma.

*DNA analysis*

The results of DNA analysis of DNA ploidy content of the 178 cases are summarized in Table I. The mean half peak coefficient of variation of diploid cases was 6.4% as measured by standard software (Coulter Electronics, Hialeh, Florida, USA). Forty-seven of 178 (26%) of cases showed evidence of DNA aneuploidy. The greatest number of DNA aneuploid cases was observed among high grade lymphomas (27/72; 38%) with significantly fewer cases (20/106; 19%) occuring in low grade lymphomas ($P<0.01$). There was no significant association between sex and age of patient, or nodal and extranodal status of lymphoma and ploidy values. Analysis of the DNA index of DNA aneuploid cases revealed a bimodal distribution of DNA content with a tendency to cluster around DNA indices of 1.3 and 2 (Figure 2).

*Proliferative index*

The distribution of proliferative index among lymphomas is shown in Figure 3. Low grade lymphomas had lower mean values (15.7%) than high grade tumours (24.24%). The mean proliferative index of all 178 cases was 19%. Cases with a proliferative index of 19% and above were found significantly more often among high grade tumours than among low grade tumours ($P<0.001$).

*Survival*

The relationship of morphological grade of lymphoma, ploidy status, DNA index and proliferative index to survival was assessed in the cases diagnosed during 1963–67. The survival data was absolute and not corrected for age. The association between morphological grade of NHL and survival assessed using long rank statistics over the whole 20 yr period approached significance ($P=0.1$) (Figure 4). Assessment at 3 yr and 7 yr respectively using $\chi^2$ test revealed significant results at 3 yr ($P=0.01$) but not at 7 yr ($P=0.07$). Neither ploidy, DNA index nor proliferative index were associated with survival (see Figures 5 and 6) when assessed over the whole 20 yr period (log rank test) or in the short term (3 yr; $\chi^2$ test). The latter three variables still gave non-significant differences in survival when controlled for grade using the log rank test even when variable proliferative indices were employed as described by Roos *et al.* (1985).

*Clinical remission and ploidy*

The relationship between DNA content of the 23 high grade tumours diagnosed during 1980–85 and the achievement of complete clinical remission was assessed. Whilst complete remission was obtained in 6/6 (100%) DNA aneuploid cases only 7/17 (41%) of the diploid cases achieved this result ($P<0.05$).

**Figure 2** Distribution of ploidy indices (DNA index) among DNA aneuploid cases.

**Figure 3** Distribution of proliferative index (% cells in $S+G_2$) in high and low grade tumours, —— mean value.

Analysis of the difference in remission rate between high grade lymphomas with high and low proliferative indices failed to reach statistical significance. There was insufficient follow up time to analyse the 1980–85 data for association with survival.

### Discussion

Although a number of workers have documented the relationship between the morphological grade of NHL and DNA content (Shackney *et al.*, 1984; Braylan *et al.*, 1984), no previous study has been able to determine the significance of differences in DNA content in relation to long term survival. In this study we have shown that statistically significant differences in frequency of DNA aneuploidy between low and high grade lymphomas can be demonstrated using paraffin embedded material. However, the present study has failed to demonstrate that assessment of ploidy status provides a better prognostic indicator than morphological grade. Indeed, the survival curves reveal no differences between DNA aneuploid and diploid tumours, even when differences in grade were taken into account. Concerning analysis of survival of NHL according to grade, this only approached significance when assessed over 20 yr, however survival at 3 yr proved significant. The survival curves according to grade (Figure 4) show great similarity to those produced by workers in

**Figure 4** Cumulative survival curve according to grade of non-Hodgkin's lymphoma. (——), high grade; (------), low grade.

**Figure 5** Cumulative survival curve of DNA aneuploid and diploid tumours. (------), aneuploid; (——), diploid.

**Figure 6** Cumulative survival curve in relation to proliferative index above or below 20%. (——) >20%; (------), ≦20%.

Kiel (Brittinger *et al.*, 1984). However the low and high grade curves merge at a later stage in the present study. This probably reflects differences in therapeutic regimens.

It should be realized the retrospective nature of the study precluded evaluation of the influence of such factors as stage of lymphoma and heterogeneity of DNA content within individual tumours. It will be necessary to consider these factors in prospective studies.

Long term survival data was only available in the 1963–67 cases when treatment consisted largely of radiotherapy with only a small proportion of patients receiving cyclophosphamide therapy in addition. However, the observation in this study with patients from a later period (1980–85), that high grade DNA aneuploid tumours treated with modern combination chemotherapy, show a high rate of complete remission is of considerable interest. Similar findings have been made in infantile neuroblastomas (Look *et al.*, 1984) and childhood acute lymphoblastic leukaemias (Look *et al.*, 1983). Whether such enhanced susceptibility to chemotherapy will result in a more prolonged survival or freedom from relapse has yet to be determined. It is interesting to speculate that there may be a link between cell survival and the effective action of the cytotoxic agents relating to DNA content.

Measurement of DNA index in the DNA aneuploid tumours revealed a bimodal distribution confirming the findings of others (Braylan *et al.*, 1984; Shackney *et al.*, 1984). It has been suggested that some diploid tumours become DNA tetraploid with subsequent development of cytogenetic instability and chromosome loss to become DNA aneuploid tumours. The chromosome loss is believed to accompany an increase in tumour aggressiveness in keeping with the clonal evolution theory (Nowell, 1976). For this reason some workers suggest DNA tetraploid tumours may have a better prognosis than DNA aneuploid tumours (Bunn *et al.*, 1980; Isaacs *et al.*, 1982). We have not been able to confirm this suggestion in this study.

Previous investigations (Braylan *et al.*, 1984; Diamond *et al.*, 1982; Shackney *et al.*, 1984) using fresh tissue have shown that the proportion of tumour cells in S phase of the cell cycle correlates with the lymphoma morphological grade. Our results confirm this finding using reprocessed paraffin embedded material. Using a combination of $S+G_2$ as a measure of the proliferative index and a cut off point of 19% (mean value for all cases), the majority of low grade lymphomas were found to lie below this value whereas most high grade tumours were above. Recent work has suggested that in the short term (2–3 yr) proliferative index correlates with survival (Roos *et al.*, 1985). The present study does not confirm these findings over a similar period of time (3 yr) or over a longer period of time (15–20 yr). An explanation for this may lie in the different modes of therapy applied to the two groups.

The finding that the presence of DNA

aneuploidy in high grade lymphomas treated with aggressive chemotherapy is associated with a high rate of first remission may have considerable clinical implications. There is now an urgent need to follow this further to determine the importance of DNA content as regards its influence on future therapy.

This work was supported by a grant from the Yorkshire Cancer Research Campaign. We wish to express our gratitude to colleagues at York District General and Castle Hill Hospitals for permission to use cases from their laboratories. We are also indebted to Mrs C. North and Mr A. Roberts for expert technical assistance and to Mrs V.E. Binns for help with the statistical analyses.

**References**

ARMITAGE, N.C., ROBINS, R.A., EVANS, D.F., TURNER, D.R., BALDWIN, R.W. & HARDCASTLE, J.D. (1985). The influence of tumour cell DNA abnormalities on survival in colorectal cancer. *Br. J. Surg.*, **72**, 828.

BAGWELL, C.B. (1979). Ph.D. dissertation. University of Miami School of Medicine, Miami, Florida.

BIRD, C.C., LAUDER, I. KELLETT, H.S. & 5 others (1984). Yorkshire Regional Lymphoma Histopathology Panel: Analysis of five year's experience. *J. Pathol.*, **143**, 249.

BRAYLAN, R.C., BENSON, N.A. & NOURSE, V.A. (1984). Cellular DNA of human neoplastic B cells measured by flow cytometry. *Cancer Res.*, **44**, 5010.

BRITTINGER, G., BARTELS, H., COMMON, H. & 48 others (1984). Clinical and prognostic relevance of the Kiel classification of non-Hodgkin lymphomas results of a prospective multicenter study by Kiel lymphoma study group. *Haematol. Oncol.*, **2**, 269.

BUNN, P.A., WHANG-PENG, J., CARNEY, D.N., SCHLAM, M.L., KNUTSEN, T. & GAZDAR, A.F. (1980). DNA content analysis by flow cytometry and cytogenetic analysis in mycosis fungoides and Sezary syndrome. *J. Clin. Invest.*, **65**, 1440.

DIAMOND, L.W., NATHWANI, B.N. & RAPPAPORT, H. (1982). Flow cytometry in the diagnosis and classification of malignant lymphoma and leukaemia. *Cancer*, **50**, 1122.

FRIEDLANDER, M.L., HEDLEY, D.W. & TAYLOR, I.W. (1984*a*). Clinical and biological significance of aneuploidy in human tumours. *J. Clin. Pathol.*, **37**, 961.

FRIENDLANDER, M.L., HEDLEY, D.W., TAYLOR, I.W., RUSSELL, P., COATES, A.S. & TATTERSHALL, M.H.N. (1984*b*). Influence of cellular DNA content on survival in advanced ovarian cancer. *Cancer Res.*, **44**, 397.

HEDLEY, D.W., FRIEDLANDER, M.L., TAYLOR, I.W., RUGG, C.A. & MUSGROVE, E.A. (1983). Method for analysis of cellular DNA content of paraffin embedded pathological material using flow cytometry. *J. Histochem. Cytochem.*, **31**, 1333.

HIDDEMAN, W., SCHUMANN, J., ANDREEFF, M. & 6 others (1984). Convention on nomenclature for DNA cytometry. *Cytometry*, **5**, 445.

ISAACS, J.T., WAKE, N., COFFEY, D.S. & SANDBERG, A.A. (1982). Genetic instability coupled to clonal selection as a mechanism for tumour progression in the Dunning R-3327 rat prostatic adenocarcinoma system. *Cancer Res.*, **42**, 2353.

LENNERT, K. (1978). Malignant lymphomas other than Hodgkin's disease *Handbuch der Speziellen Pathologischen Anatomie und Histologie: Berlin.*

LOOK, A.T., HAYES, F.A., NITSCHKE, R., McWILLIAMS, N.B. & GREEN, A.A. (1984). Cellular DNA content as a predictor of response to chemotherapy in infants with unresectable neuroblastoma. *N. Engl. J. Med.*, **311**, 231.

LOOK, A.T., MELVIN, S.L., WILLIAMS, D.L. (1983). Clinical and biological complications of flow cytometric determination of aneuploidy and pretreatment % S phase of marrow blasts in childhood acute lymphoblastic leukaemia. Seventh Annual Meeting of the Cell Kinetics Society, **G2**, 54.

NOWELL, P.C. (1976). The clonal evolution of tumour cell populations. Acquired genetic lability permits stepwise selection of variant sublines and underlines tumour progression. *Science*, **194**, 23.

PETRO, R., PIKE, M.C., ARMITAGE, P. & 7 others (1976). Design and analysis of randomised clinical trials requiring prolonged observation of each patient. *Br. J. Cancer*, **34**, 585.

ROOS, G., DIGE, U., LENNER, P., LINDH, J. & JOHANSSON, H. (1985). Prognostic significance of DNA-analysis by flow cytometry in non-Hodgkin's lymphoma. *Haematol. Oncol.*, **3**, 233.

SHACKNÉY, S.E., LEVINE, A.M., FISHER, R.I. & 10 others. (1984). The biology of tumour growth in the non-Hodgkin's lymphomas: A dual parameter flow cytometry study of 220 cases. *J. Clin. Invest.*, **73**, 1201.

SRIGLEY, J., BARLOGIE, B., BUTLER, J.J. & 7 others (1985). Heterogeneity of non-Hodgkin's lymphoma probed by nucleic acid cytometry. *Blood*, **65**, 1090.